# Simultaneous assessment of NAD(P)H and flavins with multispectral fluorescence lifetime imaging microscopy at a single excitation wavelength of 750 nm

**DOI:** 10.1117/1.JBO.29.10.106501

**Published:** 2024-09-30

**Authors:** Boris Yakimov, Anastasia Komarova, Elena Nikonova, Artem Mozherov, Liubov Shimolina, Marina Shirmanova, Wolfgang Becker, Evgeny Shirshin, Vladislav Shcheslavskiy

**Affiliations:** a Sechenov First Moscow State Medical University, Laboratory of Clinical Biophotonics, Moscow, Russia; b M.V. Lomonosov Moscow State University, Faculty of Physics, Moscow, Russia; c Privolzhsky Research Medical University, Institute of Experimental Oncology and Biomedical Technologies, Novgorod, Russia; d Becker&Hickl GmbH, Berlin, Germany

**Keywords:** fluorescence lifetime imaging, time-correlated single photon counting, microscopy, metabolism, NAD(P)H, flavins

## Abstract

**Significance:**

Autofluorescence characteristics of the reduced nicotinamide adenine dinucleotide and oxidized flavin cofactors are important for the evaluation of the metabolic status of the cells. The approaches that involve a detailed analysis of both spectral and time characteristics of the autofluorescence signals may provide additional insights into the biochemical processes in the cells and biological tissues and facilitate the transition of spectral fluorescence lifetime imaging into clinical applications.

**Aim:**

We present the experiments on multispectral fluorescence lifetime imaging with a detailed analysis of the fluorescence decays and spectral profiles of the reduced nicotinamide adenine dinucleotide and oxidized flavin under a single excitation wavelength aimed at understanding whether the use of multispectral detection is helpful for metabolic imaging of cancer cells.

**Approach:**

We use two-photon spectral fluorescence lifetime imaging microscopy. Starting from model solutions, we switched to cell cultures treated by metabolic inhibitors and then studied the metabolism of cells within tumor spheroids.

**Results:**

The use of a multispectral detector in combination with an excitation at a single wavelength of 750 nm allows the identification of fluorescence signals from three components: free and bound NAD(P)H, and flavins based on the global fitting procedure. Multispectral data make it possible to assess not only the lifetime but also the spectral shifts of emission of flavins caused by chemical perturbations. Altogether, the informative parameters of the developed approach are the ratio of free and bound NAD(P)H amplitudes, the decay time of bound NAD(P)H, the amplitude of flavin fluorescence signal, the fluorescence decay time of flavins, and the spectral shift of the emission signal of flavins. Hence, with multispectral fluorescence lifetime imaging, we get five independent parameters, of which three are related to flavins.

**Conclusions:**

The approach to probe the metabolic state of cells in culture and spheroids using excitation at a single wavelength of 750 nm and a fluorescence time-resolved spectral detection with the consequent global analysis of the data not only simplifies image acquisition protocol but also allows to disentangle the impacts of free and bound NAD(P)H, and flavin components evaluate changes in their fluorescence parameters (emission spectra and fluorescence lifetime) upon treating cells with metabolic inhibitors and sense metabolic heterogeneity within 3D tumor spheroids.

## 1 Introduction

Fluorescence lifetime imaging (FLIM) has found multiple applications in fundamental cell biology and biomedical diagnostics. One of the main applications of FLIM is probing cellular metabolism using autofluorescence of the reduced nicotinamide adenine dinucleotide (phosphate) NAD(P)H and oxidized flavins—flavin adenine dinucleotide (FAD) and flavin mononucleotide (FMN) (hereafter referred to as flavins), which are responsible for numerous redox reactions within the cell. This approach is based on measuring the fluorescence lifetimes of NAD(P)H and flavins, which depend primarily on the binding to proteins. Generally, NAD(P)H has more intense fluorescence in cancer cells and tissues, and the interpretation of its fluorescence lifetime changes is more unambiguous compared with flavins. Therefore, NAD(P)H fluorescence is more often used for metabolic imaging in cancer research.^1^^,^^2^

Although the fluorescence kinetics of NAD(P)H and FAD/FMN is complex, their fluorescence decays can be well described by a bi-exponential function.^3^^,^^4^ The fast exponent in the NAD(P)H decay (∼0.4  ns) is attributed to its free form, associated with glycolysis, and the slow component (∼1.5 to 3.5 ns)—to enzyme-bound form, attributed to the mitochondrial respiratory chain. The fast component of the FAD decay (∼0.3  ns) is associated with the quenching of isoalloxazine emission by adenine within the molecular complex, and the slow component (∼2.5 to 3.0 ns) is attributed to the unquenched state.^1^^,^^5^ The fluorescence of FMN decays more slowly with a lifetime of ∼4.5 to 5 ns.^6^^,^^7^ The ratio of these two components of NAD(P)H and FAD decays, as well as the mean fluorescence lifetime, serves as a descriptor of changes in cellular metabolism and allows investigation of such processes as cancer progression and response to therapies, cell differentiation, and oxidative stress. Moreover, the use of NAD(P)H fluorescence decay was demonstrated to be capable of visualization of cellular metabolic heterogeneity and delineating tumor margins both *ex vivo* and *in vivo*.^8^^,^^9^ However, further development of metabolic FLIM requires software and technical advances. The software advances include more sophisticated, precise, and fast methods of FLIM data analysis, whereas the technical advances include using faster detectors and electronics, optical schemes that can improve signal-to-noise ratio, and in general, any upgrades delivering more detailed information about cells’ autofluorescence.

One of the routes toward more informative FLIM is to combine spectral information with the analysis of fluorescence decay parameters. In one of the first works on combined time and spectrally resolved measurements, the authors investigated the interplay between the signals from NAD(P)H and flavins in monolayered cell cultures.^10^ Using a bi-exponential model and decay-associated spectra (DAS), they have shown that the ratio of free and bound NAD(P)H could serve as a parameter that can differentiate normal and cancerous cells. Multi-wavelength fluorescence lifetime spectroscopy has been also applied to study endogenous fluorescence from cardiac cells.^11^^,^^12^ The combined data from time-resolved area-normalized emission spectroscopy, global analysis (DAS), and principal component analysis allowed the authors to estimate the number of spectral components after metabolic modulation of cardiac cells with a 375 nm ps laser. In a recent work, we demonstrated the possibility of precisely differentiating different fluorophores (e.g., NAD(P)H and GFP) having rather similar fluorescence lifetimes and overlapping emission spectra with the use of the spectral detector and laser multiplexing on the confocal macro-FLIM system.^13^ Here, we extended these investigations to FLIM-microscopy of cellular autofluorescence. Namely, it is known that the detection of flavins in addition to NAD(P)H typically requires sequential scanning of the sample, which increases light exposure and possible phototoxic and thermal effects on the cells. Although simultaneous excitation and detection of NAD(P)H and flavins are possible, this can result in the contamination of the fluorescence signal of flavins by NAD(P)H.^6^ Here, we present the experiments on multispectral FLIM with a detailed analysis of NAD(P)H and flavin fluorescence decays under a single excitation wavelength aimed at understanding whether the use of multispectral detection is helpful for metabolic imaging of cancer cells. Starting from model solutions, we switched to cell cultures treated by metabolic inhibitors and then studied the metabolism of cells within tumor spheroids. Compared with the previous works 8 to 10 that have been related to the time-resolved spectroscopic measurements, where all of the data have been averaged across the images, our work is focused on spectral time-resolved imaging where each cell is individually mapped. Thus, spectroscopic and fluorescence lifetime heterogeneity can be followed not only between the different cells but also inside them.

## 2 Materials and Methods

### 2.1 Solutions of NADH

Tris-buffered saline (diH20, 50 mM Tris, 150 mM NaCl) at pH 7.6 was used as the solvent for all solutions. To prepare NADH (#43420, Sigma-Aldrich, St. Louis, Missouri, United States) solution, a stock solution with a concentration of 1 mM was initially obtained, which was then diluted to 50  μM. To prepare the protein-bound form, lactate dehydrogenase (LDH) from the porcine heart (#L7525, Sigma-Aldrich) was added to the NADH (50  μM) solution at a concentration of 13  μM. The concentrations of the solutions were selected based on the literature data.^14^^,^^15^ The 100  μl drops of free and LDH-bound NADH were placed on 35 mm glass-bottomed FluoroDish (Ibidi GmbH, Gräfelfing, Germany) for test measurements.

### 2.2 Cell Lines and Spheroids

Hela Kyoto cell line (human cervical cancer) was routinely cultured in a ${\mathrm{CO}}_{2}$ incubator at 37°C in an atmosphere of 5% ${\mathrm{CO}}_{2}$, using a Dulbecco’s modified Eagle’s medium (DMEM) nutrient medium (Paneko, Russia) supplemented with 2 mM glutamine (Paneko, Russia), 10  mg/ml streptomycin (Paneko, Russia), and 10% fetal bovine serum FBS (HyClone). Hela Kyoto cells were seeded on glass bottom FluoroDish for confocal fluorescence microscopy (World Precision Instruments) at $5\times 10^{5}\text{ cells}$ per dish in FluoroBrite DMEM without phenol red (Thermo Fisher Scientific, Waltham, Massachusetts, United States). 3-Bromopyruvate (Sigma-Aldrich) and rotenone (Sigma-Aldrich) were used as inhibitors of metabolic pathways, glycolysis, and oxidative phosphorylation, correspondingly. A solution of 3-bromopyruvate at a concentration of 15  μM was added to the cells for 24 h. Rotenone at a concentration of 1  μM was added for 1 h. Cells without the inhibitors were used as a control.

For the formation of tumor spheroids, HeLa Kyoto cells were seeded on round-bottom non-adhesive 96-well plates (Corning, United Kingdom) at 500 cells per well in 200  μl of DMEM supplemented with 2 mM glutamine, 10  mg/ml streptomycin, and 10% FBS. The spheroids were cultured for 5 days in a ${\mathrm{CO}}_{2}$ incubator at 37°C, 5% ${\mathrm{CO}}_{2}$. The advanced spheroids (n=3) with a size of ≈500  μm were gently transferred to the glass-bottom dish for confocal microscopy with a Pasteur pipette, in a FluoroBrite DMEM medium supplemented with 10% FBS. FLIM was performed within the additional 2 to 3 h when the spheroids were attached to the glass bottom of the FluoroDish.

### 2.3 FLIM Setup

Multispectral fluorescence lifetime images were recorded by multi-dimensional time-correlated single photon counting.^16^^–^^18^ An LSM 880 NLO multiphoton laser scanning microscope (Carl Zeiss, Germany) was equipped with a PML-SPEC-GaAsP multi-wavelength detector and an SPC-150N TCSPC/FLIM module both from Becker & Hickl GmbH, Germany. The excitation source was a Mai-Tai femtosecond Ti:Sa laser (Spectra Physics, Milpitas, California, United States). The endogenous cofactor NAD(P)H was excited in the two-photon mode at a wavelength of 750 nm with a pulse repetition rate of 80 MHz and a pulse duration of 120 fs. The endogenous fluorescence was transferred to a PML-SPEC-GaAsP multi-wavelength detector attached to the NDD port of the microscope with a fiber bundle. Inside the detector, the fluorescence light was split spectrally by a grating and projected on the 16 channels of a multi-channel PMT.^16^ The bandwidth of each spectral channel was 12.5 nm, and the overall spectral range for the detection was from 388.1 to 573.6 nm. The temporal resolution (instrument response function) of the detector was 220 ps full-width at half maximum (FWHM) or ∼96  ps root mean square (RMS).^17^ It should be noted that when using a spectral detector, there is no need to use band-pass filters in the detection channel because the spectral selection is done by a diffraction grating. For experiments with solutions and cells, a water-immersion (C-Apochromat 63x/1.2, Zeiss) and oil-immersion (C-Apochromat 40x/1.4, Zeiss) objective lenses were used. The size of the field of view was 212×212  μm corresponding to 1024×1024  pixels images.

### 2.4 Cell Segmentation Procedure

For cell segmentation, we used an automatic segmentation algorithm previously described in Ref. 6. Briefly, a hybrid approach utilizing a convolutional neural network with the U-net such as architecture and classical computer vision post-processing algorithms was implemented to predict cells’ boundaries, cytoplasm, and nuclei.

First, the intensity images were normalized to the [−0.5,0.5] range to match the inputs of the U-net neural network. Then, the preprocessed images served as an input of the multiple U-nets, which predicted cells’ borders, inner regions of cells, and cells’ nuclei, i.e., each U-net for a given image predicted a map of probabilities for pixels to belong to a cell’s border, cytoplasm, or nucleus. The obtained probability maps were then thresholded and post-processed using morphological operations to remove artifacts. The probability maps of cells’ nuclei were used to compute the coordinates of the nuclei centroids. The processed binary masks and positions of the nuclei centroids were used to calculate distance maps from the nuclei centers to the cells’ borders. The obtained distance maps were further subject to the watershed algorithm, which returned the final masks.

The fluorescence signal was integrated over the entire segmented area of each cell and used for further analysis. In total, five fields of view for the control sample, four fields of view of the cells exposed to rotenone, and five fields of view for the cells, exposed to 3BP, were obtained, where, in total, 754 cell regions were analyzed (251 control cells, 285 cells incubated with rotenone, and 218 cells incubated with 3BP).

### 2.5 Fluorescence Decay Fitting Procedures

We employed two fitting procedures to analyze the fluorescence decay parameters of the fluorescence decay curves obtained at different emission wavelengths. In the first procedure, each spectral channel was analyzed individually using a bi-exponential decay law model with a convolution with the instrument response function (IRF(t)), assuming that IRF is modeled by a Gaussian function

$$F(t)∼\mathrm{IRF}(t)⊗(a_{1}\;\exp(-\frac{t}{\tau_{1}})+a_{2}\;\exp(-\frac{t}{\tau_{2}})),$$ (1)

where $a_{i}$, $\tau_{i}\;(i=1,2)$ are the amplitudes and lifetimes of the i’th component. We also calculated the values of the mean fluorescence lifetime $\tau_{m}$

$$\tau_{m}=\frac{a_{1}\tau_{1}+a_{2}\tau_{2}}{a_{1}+a_{2}}.$$

The second approach involved a model that considered both temporal and spectral features of the detected fluorescent response. The fluorescence response F(λ,t) observed at multiple emission wavelengths λ for different times t after the start of excitation was fitted using a tri-exponential model in which the amplitudes of the fluorescence decay kinetics $a_{i}(\lambda )$ were dependent on the emission wavelength, whereas the fluorescence lifetimes $\tau_{i}$ were shared across all spectral channels within each of the three components:

$$F(\lambda ,t)∼\mathrm{IRF}(t)⊗\overset{3s}{\underset{i=1}{\sum }}a_{i}(\lambda )\exp\;\exp(-\frac{t}{\tau_{i}}).$$ (2)

We attributed different components to the different fluorophores by introducing additional constraints into the model. The shape of emission spectra of the first two components $a_{1}(\lambda )$ and $a_{2}(\lambda )$ was fixed to the spectra obtained for free and bound NADH solutions obtained experimentally (i.e., $a_{\text{free}}^{\mathrm{NAD}(P)H}(\lambda )\equiv a_{1}(\lambda )$ and $a_{\text{bound}}^{\mathrm{NAD}(P)H}(\lambda )\equiv a_{2}(\lambda )$), whereas amplitudes of the components could vary. The decay time of the first component was fixed to the fluorescence decay lifetime of the free NADH ($\tau_{\text{free}}^{\mathrm{NAD}(P)H}\equiv \tau_{1}=0.37\;\mathrm{ns}$) and was not a fitting parameter. We could fix the fluorescence decay time of free NAD(P)H as it does not vary significantly from one experiment to another compared with the fluorescence decay times of the bound form in most of the previous canonical FLIM studies.^2^^,^^19^^,^^21^

The decay time of the second component attributed to bound NADH ($\tau_{\text{bound}}^{\mathrm{NAD}(P)H}\equiv \tau_{2}$) was determined by fitting to experimental data. We constrained the range for $\tau_{\text{bound}}^{\mathrm{NAD}(P)H}$ value between 0.5 and 10 ns to enhance the reliability of the fitting results, as typical values for the fluorescence decay time of bound NAD(P)H are within the 1 to 5 ns range.^21^

To account for flavin contributions to the fluorescence decay kinetics, a third component with a decay time $\tau_{\text{flavins}}\equiv \tau_{3}$ was included, with the value obtained by fitting to experimental the other components, the emission spectrum of flavins was not fixed, allowing the decay amplitudes $a_{3}(\lambda )$ to vary independently across spectral channels. In addition, the amplitudes of this component were fixed to zero at wavelengths less than 490 nm: $a_{3}(\lambda <490\;\mathrm{nm})\equiv 0$.

Finally, to evaluate the relative contribution of free NADH, bound NADH, and flavins, we estimated the maximal value of each of the components across all spectral channels $A_{1}=\max_{\lambda }\;a_{1}(\lambda )$, $A_{2}=\max_{\lambda }\;a_{2}(\lambda )$, $A_{3}=\max_{\lambda }\;a_{3}(\lambda )$, and then introduced values $a_{\text{free}}^{\mathrm{NAD}(P)H}$, $a_{\text{bound}}^{\mathrm{NAD}(P)H}$, and $a_{\text{flavins}}$ calculated as [Eq. (3)]

$$a_{\text{free}}^{\mathrm{NAD}(P)H}=\frac{A_{1}}{A_{1}+A_{2}+A_{3}},\;a_{\text{bound}}^{\mathrm{NAD}(P)H}=\frac{A_{2}}{A_{1}+A_{2}+A_{3}},\;a_{\text{flavins}}=\frac{A_{3}}{A_{1}+A_{2}+A_{3}}.$$ (3)

In addition, to characterize NAD(P)H fluorescence decay, we calculated the mean fluorescence lifetime $\tau_{m}^{\mathrm{NAD}(P)H}$ as [Eq. (4)]

$$\tau_{m}^{\mathrm{NAD}(P)H}=\frac{a_{\text{free}}^{\mathrm{NAD}(P)H}*\tau_{\text{free}}^{\mathrm{NAD}(P)H}+a_{\text{bound}}^{\mathrm{NAD}(P)H}*\tau_{\text{bound}}^{\mathrm{NAD}(P)H}}{a_{\text{free}}^{\mathrm{NAD}(P)H}+a_{\text{bound}}^{\mathrm{NAD}(P)H}}.$$ (4)

All processing was implemented in Python 3.10 using NumPy, Pandas, Matplotlib, Scikit-Learn, and Lmfit libraries. Spatial binning was equal to 5, i.e., an 11×11 sliding window was used to obtain a decay curve. To visualize the spectral differences in the fluorescence response, we mapped the average intensity-weighted fluorescence emission wavelength calculated as $\lambda_{\mathrm{avg}}=\sum \lambda_{i}I_{i}/\sum I_{i}$ using the intensity $I_{i}$ in the i’th spectral channel, which corresponds to the emission wavelength $\lambda_{i}$.

## 3 Results

### 3.1 Free and Bound NADH in Model Solutions

We first analyzed the differences in the spectral and time-resolved responses using model solutions of pure NADH in Tris-buffered saline (the free form) and a mixture of NADH and LDH (the protein-bound form) upon two-photon excitation at 750 nm in 16 emission channels, which covers 380 to 580 nm range with ∼12.5  nm resolution.

**Fig. 1:**
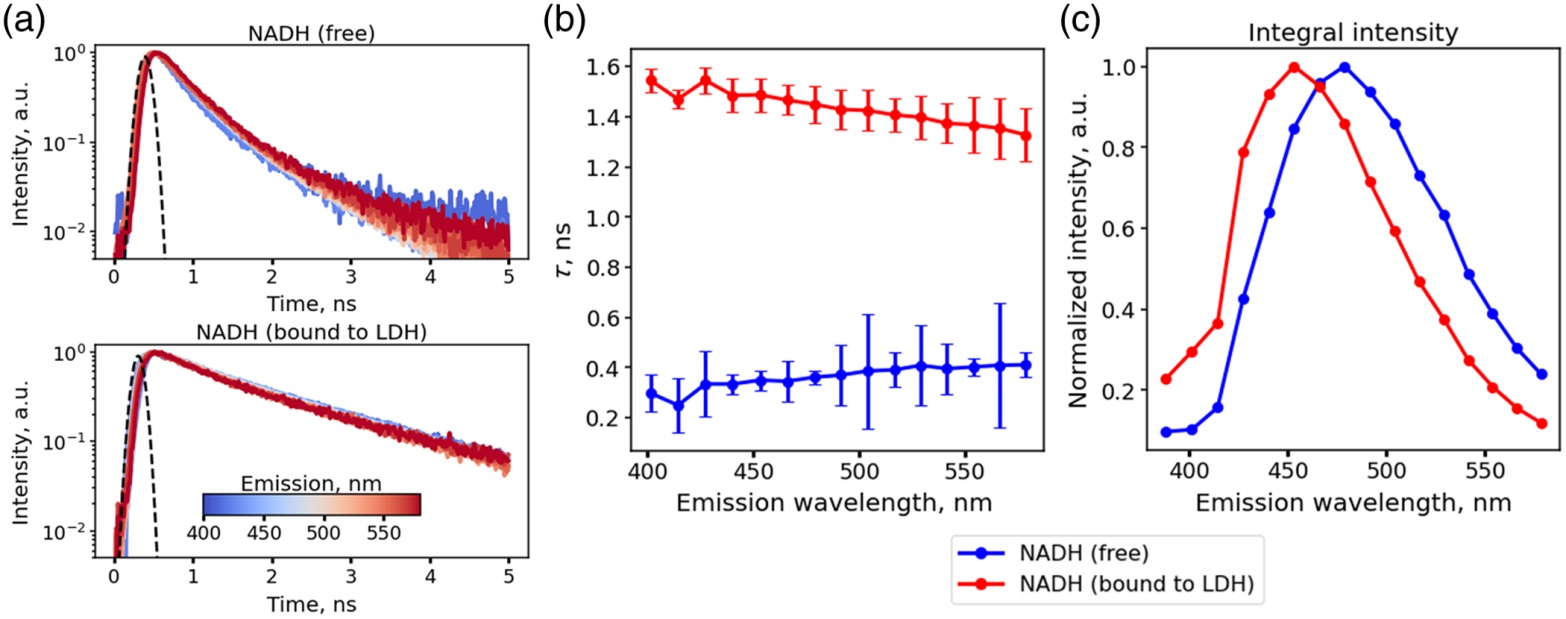
Time-resolved multispectral data on free and bound NAD(P)H in solution. (a) Fluorescence decay curves of free (blue) and LDH-bound (red) NADH in the emission range of 380 to 580 nm. (b) Mean fluorescence decay times. (c) Time-averaged (i.e., steady-state) fluorescence emission spectra for free and LDH-bound NADH in solutions.

As expected, fluorescence decay curves of free NADH demonstrated faster fluorescence decay compared with LDH-bound NADH, whereas the decays of both samples were weakly dependent on the fluorescence emission wavelength in the used emission range [Fig. 1(a)]. In all emission channels, the fluorescence decay curve of free NADH was well described by a bi-exponential decay with characteristic lifetimes of $\tau_{1}=0.36\pm 0.05\;\mathrm{ns}$ and $\tau_{2}=0.89\pm 0.11\;\mathrm{ns}$ and amplitudes of $a_{1}=80\pm 4\%$ and $a_{2\;}=20\pm 4\%$. The observed bi-exponential behavior may be the result of the different charge distributions in the cis and trans configurations of the NADH molecule causing different internal electrostatic field distributions.^19^ Yet, the observed values of the fast fluorescence decay time $\tau_{1}=0.36\pm 0.05\;\mathrm{ns}$ and mean fluorescence decay time $\tau_{m}=0.47\pm 0.1\;\mathrm{ns}$ were close to the values reported in the literature.^3^^,^^19^

The fluorescence decay curves of LDH-bound NADH were well described by a monoexponential fluorescence decay curve with a mean decay time of 1.44±0.07  ns [Fig. 1(a)]. Spectral dependences of the fluorescence decay time of LDH-bound NADH and the short decay component of free NADH are shown in Fig. 1(b). We observed a slight trend in the dependence of the fluorescence decay time on the emission wavelength—a decrease in the fluorescence decay time for bound NADH and an increase in the fluorescence decay time of free NADH with emission wavelength. The time-averaged fluorescence emission spectrum of the LDH-bound NADH was shifted to the short wavelength region [Fig. 1(c)]. The fluorescence emission maximum of the LDH-bound NADH was observed at ∼450  nm, in contrast to the emission maximum of free NADH (∼475  nm). The slight spectral shift to the short wavelengths of the fluorescence signal from bound NADH is known and discussed in several publications, both for the NADH solutions and in cells.^20^^,^^21^

### 3.2 Cancer Cells Treatment with Metabolic Inhibitors: Spectral Unmixing of the FLIM Data and Observation of Flavins

Next, we studied the spectral and time-resolved parameters of living HeLa cells under standard cultivation conditions and upon exposure to metabolic inhibitors 3-bromopyruvate (3BP) and rotenone using the multispectral FLIM with 750 nm excitation.

**Fig. 2:**
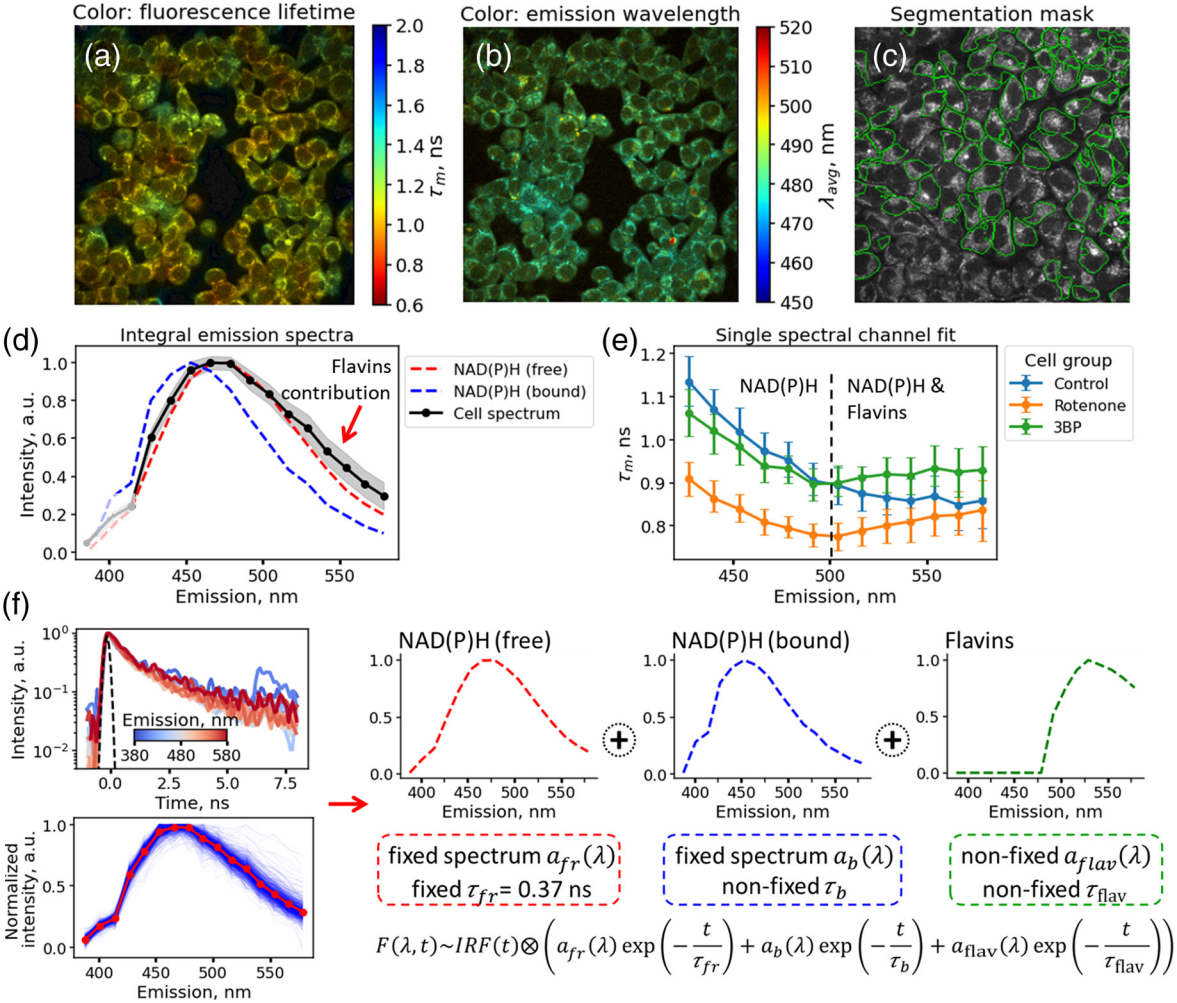
Multispectral FLIM of HeLa cells. (a), (b) Representative maps of the fluorescence lifetime $\tau_{m}$ in the NAD(P)H spectral range 475 to 485 nm (a) and the average intensity-weighted fluorescence emission wavelength $\lambda_{\mathrm{avg}}$ (b) for the control cells. (c) An example of cell segmentation masks (individual cells are circled in green). (d) Emission spectra of free (red line) and bound (blue line) NAD(P)H, as well as the time-averaged emission spectrum averaged over all cells (black line). The gray corridor reflects the interquartile range of the normalized emission spectra. (e) Dependence of the mean fluorescence lifetime $\tau_{m}$ obtained as a result of the bi-exponential fit of the fluorescence decay curve in each emission channel individually. Dots mark the median values of the fluorescence lifetime for the cells of the control, rotenone, and 3BP groups, and the error bars indicate the interquartile range of $\tau_{m}$. (f) Schematic representation of the model used to globally fit fluorescence decay curves obtained at multiple wavelengths.

As a preliminary step, we analyzed fluorescence decay curves in each spectral channel individually using a bi-exponential decay model. As can be seen in Figs. 2(a) and 2(b), both the mean fluorescence decay time $\tau_{m}$ of NAD(P)H (spectral channel 475 to 485 nm) and the weighted average fluorescence emission wavelength $\lambda_{\mathrm{avg}}$ are non-homogeneously distributed within individual and between cells, indicating that information about the spectral response can carry additional insights into the cells’ metabolic status.

For all three types of the samples (control, 3BP-, and rotenone treated), we observed that the spectral and time-resolved fluorescence response could not be described solely by the presence of free and protein-bound NAD(P)H. Namely, the emission spectra of cells demonstrated an increased intensity in the long wavelength range [>500  nm, Fig. 2(d)], whereas the dependence of the fluorescence decay time on the emission wavelength exhibited a non-monotonous dependence in cells treated with rotenone and 3BP [Fig. 2(e)]. In the case of NAD(P)H, the most red-shifted spectra would be observed for free NAD(P)H [red spectrum in Fig. 2(d)]. The presence of enzyme-bound fraction would shift the spectra to the blue range. However, the fluorescence spectra of cells exhibited the red edge above the free NAD(P)H spectrum [gray corridor in Fig. 2(d)]. Moreover, the mean lifetime should decrease with the wavelength if only free and bound NAD(P)H are present in the system: the longer the emission wavelength, the higher the contribution of the free NAD(P)H characterized by fast (0.37 ns) decay. But, again, this was not the case for cells [Fig. 2(e)].

Thus, we supposed that the fluorescence response in the long wavelength (>500  nm) region is due to the presence of flavins, which can also be two-photon excited at 750 nm and have emission at wavelengths >490  nm with a maximum of 535 nm.^22^ This possibility was previously investigated experimentally where the ratio of intensity contributions of NAD(P)H and flavins was assessed at different excitation wavelengths.^23^

To simultaneously account for spectral and time-resolved fluorescence properties and correctly disentangle the contributions of free NAD(P)H, bound NAD(P)H, and flavins, we used the global fitting model summarized in Fig. 2(f). The fluorescence decay curves in all spectral channels were simultaneously fitted by the sum of free NAD(P)H, bound NAD(P)H, and flavins, assuming that the spectra of free and bound NAD(P)H are fixed and correspond to the spectra presented in Fig. 1(c), whereas the spectrum of flavins can be non-zero only in the range of >490  nm. Each component (free NAD(P)H, bound NAD(P)H, and flavins) in the considered model can decay with its own time shared among different emission wavelengths. The decay time of free NAD(P)H was fixed to 0.37 ns,^2^^,^^20^^,^^21^ whereas the lifetimes of bound NAD(P)H and flavins were fitted from the experimental data. This model was sufficient to describe both the spectral features and the fluorescence decay in the spectral range of 380 to 580 nm. The examples of decay curves, corresponding best fits, and best model parameters are shown in Fig. S1 and Tables S1 and S2 in the Supplementary Material.

**Fig. 3:**
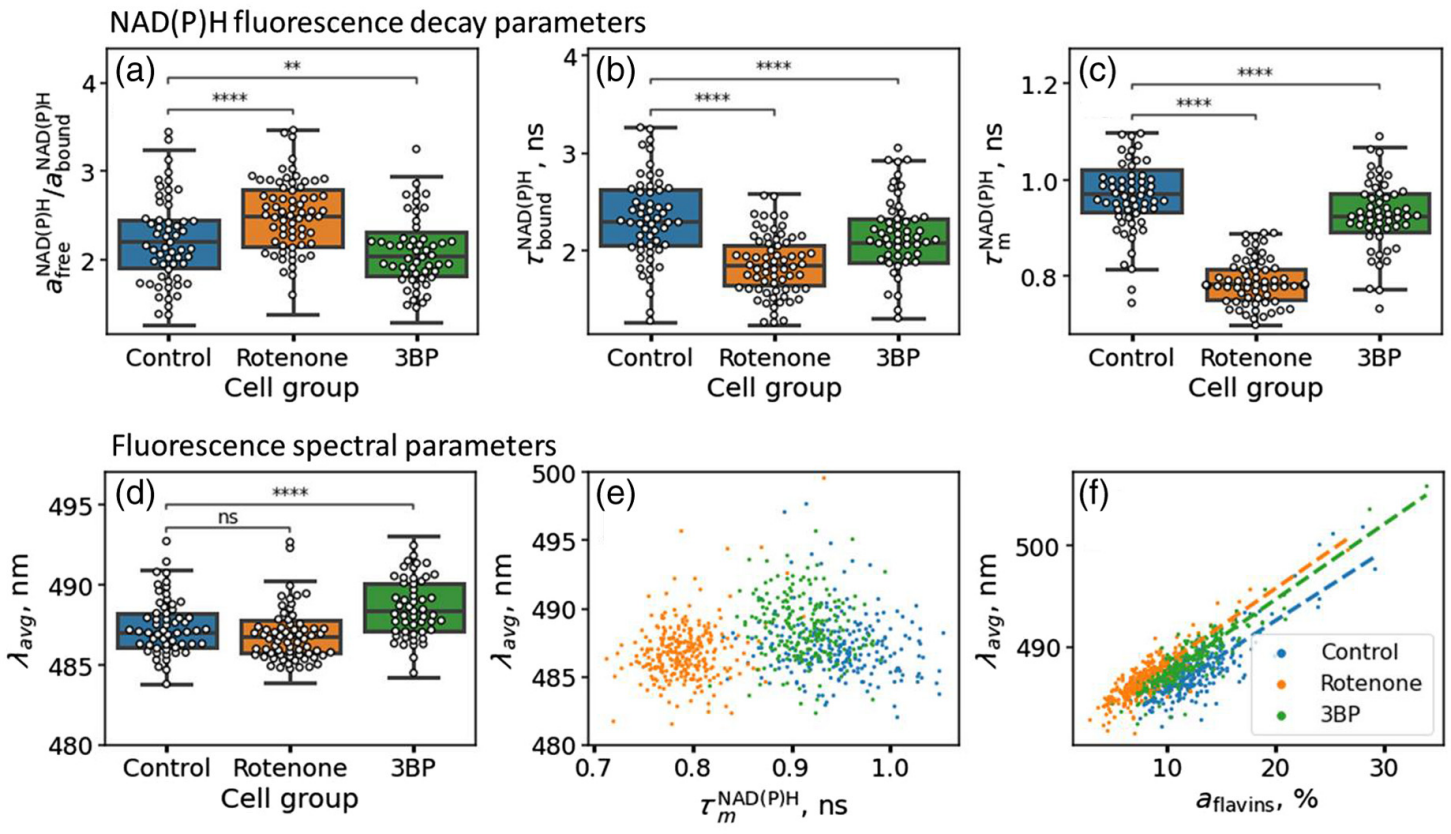
NAD(P)H fluorescence decay and spectral parameters in HeLa cells assessed from multispectral FLIM data. (a) Boxplot of the ratio of the free and bound amplitudes $a_{\text{free}}^{\mathrm{NAD}(P)H}/a_{\text{bound}}^{\mathrm{NAD}(P)H}$. (b) Boxplot of the fluorescence decay time of bound NAD(P)H: $\tau_{\text{bound}}^{\mathrm{NAD}(P)H}$; (c) Boxplot of the mean NAD(P)H fluorescence decay time $\tau_{m}^{\mathrm{NAD}(P)H}$; (d) Boxplot of the intensity-weighted average fluorescence emission wavelength $\lambda_{\mathrm{avg}}$; (e) Correlation of the $\lambda_{\mathrm{avg}}$ parameter with the mean NAD(P)H fluorescence decay time $\tau_{m}^{\mathrm{NAD}(P)H}$; (f) Correlation of the $\lambda_{\mathrm{avg}}$ parameter amplitude of the contribution of flavins to the fluorescence emission spectrum $a_{\text{flavins}}$. Dashed lines in panel (e) correspond to linear regression lines for different cells’ populations.

The developed model was employed to assess changes in the fluorescence response of cells exposed to rotenone and 3BP. Rotenone acts as a strong inhibitor of complex I of the mitochondrial respiratory chain, which leads to the blockade of oxidative phosphorylation in cells, which, in turn, should lead to an increase in the ratio of free to bound NAD(P)H^2^, $a_{\text{free}}^{\mathrm{NAD}(P)H}/a_{\text{bound}}^{\mathrm{NAD}(P)H}$. Indeed, the median value of the amplitude ratio increased from 2.7 in the control group to 3.3 in the group of cells exposed to rotenone [$p<10^{-4}$, Fig. 3(a)]. Exposure to rotenone also reduced the fluorescence decay time of bound NAD(P)H [from ∼2.5 to ∼2.1  ns, $p<10^{-4}$, Fig. 3(b)]. In combination with the increase of the $a_{\text{free}}^{\mathrm{NAD}(P)H}/a_{\text{bound}}^{\mathrm{NAD}(P)H}$ ratio, this led to a significant decrease in the mean fluorescence lifetime of NAD(P)H [0.98 versus 0.78 ns, $p<10^{-4}$, Fig. 3(c)].

3BP is an analog of pyruvic acid that acts on cellular metabolism by inhibiting hexokinase II (HK2) of the glycolytic pathway.^24^ As anticipated, the incubation of cells with 3BP resulted in a decrease in the $a_{\text{free}}^{\mathrm{NAD}(P)H}/a_{\text{bound}}^{\mathrm{NAD}(P)H}$ ratio in comparison with the control group from ∼2.7 to ∼2.65 [p<0.01, Fig. 3(a)]. A decrease in the fluorescence lifetime of the bound NAD(P)H from ∼2.5 to ∼2.35  ns was also observed [$p<10^{-4}$, Fig. 3(b)], which led to the decrease in the mean fluorescence decay time of NAD(P)H [0.92 versus 0.98 ns, $p<10^{-4}$, Fig. 3(c)].

Remarkably, the spectral properties of the overall fluorescence band were weakly correlated with changes in the time-resolved response of NAD(P)H. The cell-wise–calculated intensity-weighted average emission wavelength $\lambda_{\mathrm{avg}}$ changed only for cells exposed to 3BP and did not change for cells exposed to rotenone as compared with the control group [Fig. 3(d)]. The emission wavelength $\lambda_{\mathrm{avg}}$ weakly correlated with the fluorescence decay time $\tau_{m}^{\mathrm{NAD}(P)H}$ [Fig. 3(e), r=0.11, p<0.01] and correlated well with the relative contribution of flavins $a_{\text{flavins}}$ [Fig. 3(f), r=0.78, $p<10^{-9}$]. This indicates that the spectral changes in the case of 3BP treatment resulted from the increased contribution of flavins in cellular autofluorescence due to the shift to more oxidative metabolism, which was not revealed by the analysis of the NAD(P)H lifetime components.

We also verified that spectral-resolved microscopy without time resolution provides less information in comparison with multispectral FLIM. For this, we compared the decomposition of the time-averaged fluorescence emission spectra obtained via non-negative matrix factorization with the decomposition of the time-resolved fluorescence emission. Details are provided in Fig. S2 in the Supplementary Material.

### 3.3 Multispectral FLIM at 750 nm Excitation Reveals Alterations in Flavin Fluorescence

**Fig. 4:**
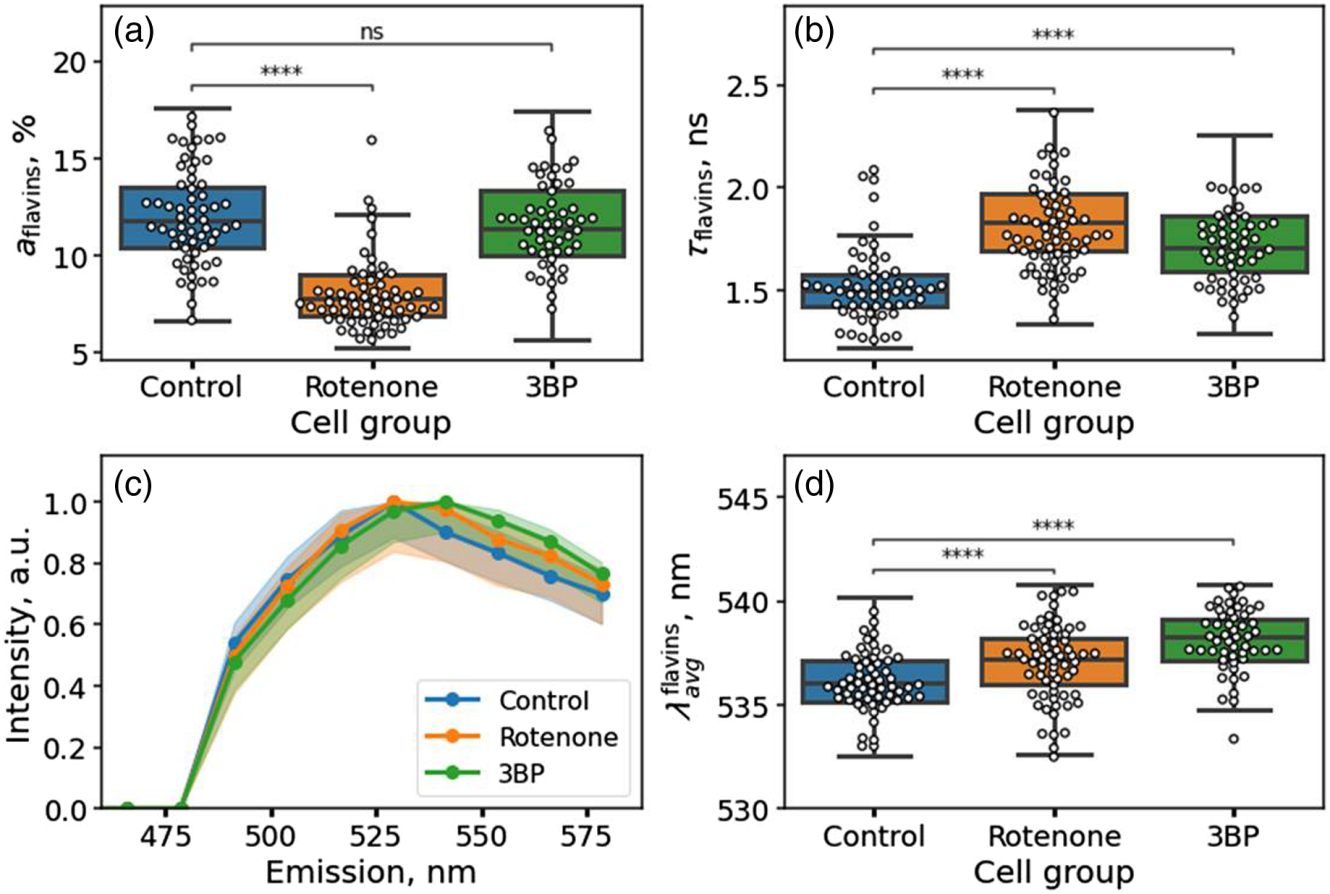
Flavin fluorescence decay parameters in HeLa cells assessed from multispectral FLIM data. (a), (b) Parameters of the fluorescence decay of the component associated with flavins for cells of the control sample, and cells exposed to rotenone and 3BP: amplitude of the flavin component $a_{\text{flavins}}$ (a) and decay time $\tau_{\text{flavins}}$ (b). (c) Median emission spectra of the component associated with flavin fluorescence in the control group of cells and cells incubated with rotenone and 3BP. (d) Distribution of the intensity-weighted average emission wavelength of the component associated with flavins $\lambda_{\mathrm{avg}}^{\text{flavins}}$ for cells of the control group and cells incubated with rotenone and 3BP.

As the next step, we analyzed fluorescence decay parameters and spectral features of the component attributed to flavins upon treatment of cancer cells with metabolic inhibitors. It was found that the normalized amplitude of flavins in the total fluorescence response signal decreased in cells exposed to rotenone (∼7.6% versus 12% in the control group, $p<10^{-4}$) and did not change for cells exposed to 3BP (∼11.5%, p>0.05) compared with control cells [Fig. 4(a)]. Statistically significant differences were also observed in the fluorescence decay times of the flavins: in the control group, the mean fluorescence decay time $\tau_{\text{flavins}}$ was equal to 1.48 ns, in the rotenone group is 1.85 ns ($p<10^{-4}$), and in the 3BP group is 1.72 ns ($p<10^{-4}$). The spectra of the component corresponding to flavins were different for the control, rotenone, and 3BP groups—the median spectra were shifted to the longer wavelength region for cells incubated with respiratory inhibitors compared with the control [Fig. 4(c)], as characterized by the intensity-weighted averaged fluorescence emission wavelength [Fig. 4(d)]. Thus, it can be concluded that the applied global fitting of the spectral and time-resolved data makes it possible not only to isolate the contribution of flavins from the integral spectra of autofluorescence but also to analyze alterations in their spectral properties with a change in the metabolic status of the cell.

### 3.4 Metabolic FLIM and Spectral Unmixing in Spheroids

Finally, spectral and time-resolved data were used to analyze metabolism within spheroids formed from HeLa cells. Multicellular three-dimensional (3D) spheroids are known to be spatially heterogeneous in their proliferative properties. The cells of the outer layers typically proliferate faster and have glycolytic (anaerobic) metabolism, whereas the cells inside the spheroid are quiescent, and their metabolism is more oxidative, although it depends on the cell type.^25^^,^^26^

**Fig. 5:**
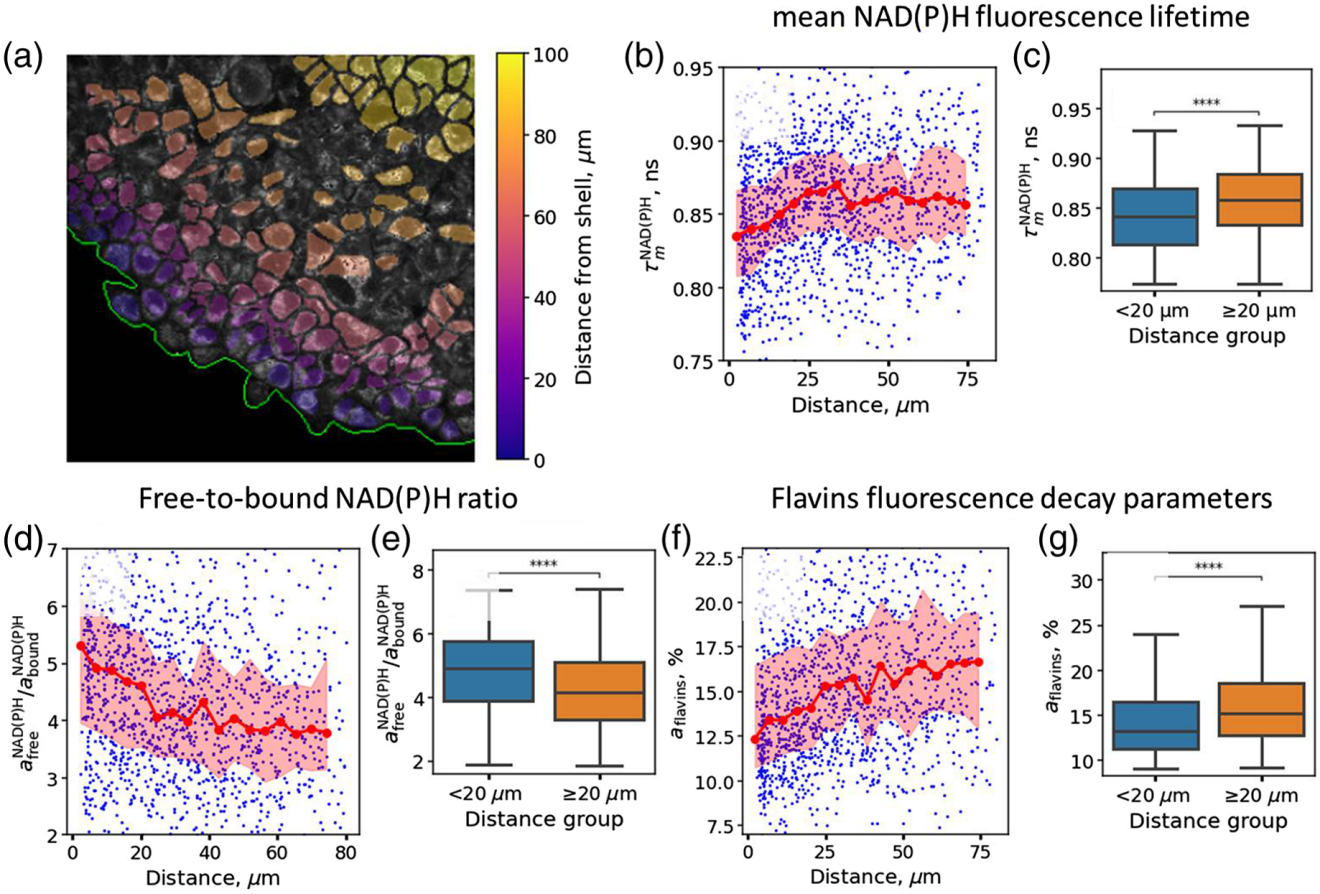
Multispectral FLIM of HeLa tumor spheroids. (a) Representative intensity image of the spheroid (gray) and masks used for segmentation, colored depending on the distance from the border of the spheroid. (b)–(g) Distribution of fluorescence decay parameters depending on the distance to the spheroid border. (b), (c) Mean fluorescence lifetime of NAD(P)H $\tau_{m}^{\mathrm{NAD}(P)H}$; (d), (e) Ratio of the amplitudes of free and bound NAD(P)H $a_{\text{free}}^{\mathrm{NAD}(P)H}/a_{\text{bound}}^{\mathrm{NAD}(P)H}$; (f), (g) Flavin amplitude $a_{\text{flavins}}$.

We automatically segmented the cells. An example of the segmentation masks, colored depending on the distance to the spheroid border, is shown in Fig. 5(a). Segmentation allowed us to simultaneously obtain information from single cells depending on their distance from the spheroid border. In total, as a result of segmentation, ∼1400 cells were isolated, located at a distance from 0 to 80  μm from the border of the spheroid. Using this segmentation mask, the spatial distribution of the fluorescence decay parameters obtained for cells inside spheroids was analyzed by the global fitting procedure described above. We observed that the mean fluorescence lifetime of NAD(P)H increased with the distance from the shell to the core of the spheroid from 0.84 to 0.86 ns [Figs. 5(b) and 5(c)], whereas the free/bound ratio $a_{\text{free}}^{\mathrm{NAD}(P)H}/a_{\text{bound}}^{NAD(P)H}$ decreased from ∼5.3 to ∼4 [Figs. 5(d) and 5(e)], which is consistent with our previous data.^24^ At the same time, we did not observe a significant trend in the dependence of the fluorescence decay time of bound NAD(P)H on the distance to the boundary (Fig. S3A in the Supplementary Material). The contribution of flavins $a_{\text{flavins}}$ to the fluorescence emission spectrum was lower in the spheroid shell compared with the core—12.5% versus 16% [Figs. 5(e) and 5(f)], additionally confirming the more oxidative metabolism of the cells inside the spheroids. The fluorescence lifetime of flavins slightly decreased upon increasing the distance from the spheroid shell (Fig. S3B in the Supplementary Material).

## 4 Discussion

Optical metabolic imaging with FLIM is widely used for studying cell biophysics and for biomedical diagnostics. Most experiments are done by using the signal from NAD(P)H as it is usually stronger and easier to interpret compared with the flavin signal. On the other hand, numerous research works demonstrated that using the flavin fluorescence solely or together with NAD(P)H benefits the sensitivity of metabolic FLIM.^26^^–^^29^ To get both of the signals (NAD(P)H and flavins), one has to do either a sequential measurement of the same sample with two excitation wavelengths (typically 375 and 445 nm for one-photon, or 750 and 900 nm for two-photon excitation) or to perform laser wavelength multiplexing.^11^^,^^26^^,^^27^ For the ps diode lasers, it can be easily realized, whereas for typical Ti:sapphire fs lasers, it is not possible to switch the wavelengths quickly. A compromise in two-photon excitation would be to set the excitation to the spectral “gap” between the absorption maxima of NAD(P)H and flavins and select bandpass filters for each of the two detectors based on certain assumptions on the spectral properties of these cofactors.^23^ Namely, the 800 nm excitation is suggested as an optimum, although the NAD(P)H signal at this excitation is almost an order of magnitude lower than that obtained at standard 730 to 760 nm wavelengths range.

Alternatively, one can use a single excitation at 750 nm for the excitation of both of the co-factors. However, although the NAD(P)H signal can be analyzed easily, the flavin signal will be contaminated with the signal from NAD(P)H. The use of the multi-channel (spectral) single photon counting detector may be a way to handle the above issue. So far, spectral FLIM microscopy has been intensively developed for the last two decades because it has high potential in biological studies.^30^^–^^34^ Spectral unmixing combined with fluorescence lifetime data can probe biochemical reactions.^35^ However, although there has been a lot of research in this field, the use of spectral FLIM microscopy for simultaneous NAD(P)H and flavin study in cancer research has been largely unexploited. There have been only a few publications related to spectral unmixing of NAD(P)H and FAD fluorescence components recorded by multi-wavelength fluorescence lifetime spectroscopy in living cardiac cells.^11^^,^^12^

In this paper, we demonstrate that the use of a multispectral detector in combination with excitation at a single wavelength of 750 nm allows the identification of fluorescence signals from three components: free and bound NAD(P)H, and flavins based on the global fitting procedure. We also investigate how the changes in the metabolic status of the cells upon their treatment with rotenone and 3-bromopyruvate influence the spectral FLIM data. Specifically, multispectral data make it possible to assess not only the lifetime but also the spectral shifts of emission of flavins caused by the chemical perturbations. Altogether, the informative parameters of the developed approach are the ratio of free and bound NAD(P)H amplitudes, the decay time of bound NAD(P)H, the amplitude of flavin fluorescence signal, the fluorescence decay time of flavins, and the spectral shift of the emission signal of flavins. Hence, with multispectral FLIM, we get five independent parameters, of which three are related to flavins. Compared with NAD(P)H, the interpretation of flavin fluorescence decay parameters is more complex and not completely clear so far. Published studies related to FLIM of flavins are quite controversial. For example, it has been reported that short-lifetime (quenched or “bound”) FAD fraction decreases with activation of glycolysis,^36^ increases with increased oxidative metabolism,^37^ or increases with increased glycolytic metabolism.^7^ In our study, the mean fluorescence decay time of flavins increased upon suppression of either mitochondrial respiration with rotenone or glycolysis with 3BP. A possible explanation for this effect is the increased contribution of FMN to the overall fluorescence of flavins. Rotenone disrupts the electron transfer from the iron-sulfur centers in complex I to ubiquinone, thus increasing the NADH level and decreasing the NAD+/NADH ratio. 3BP inhibits HK2, the enzyme that catalyzes the phosphorylation of glucose to glucose-6-phosphate, the first irreversible step of glycolysis; consequently, the cellular levels of NADH and succinate that feed electrons into complexes I and II, respectively, decrease. As a result, in the absence of electron donor (NADH) and efficient electron flow in the chain, FMN, which is a primary acceptor of electrons from NADH within complex I, is not reduced to FMNH2. Another possible reason for the longer lifetime of flavins is the diminished activity of FAD-dependent enzymes involved in the reactions of aerobic respiration, such as lipoamide dehydrogenase and succinate dehydrogenase. As fluorescence of FAD is quenched within the enzyme complexes, inactivation or decrease of the level of enzymes can result in the increased proportion of the long-lifetime fraction of flavins. In the study by Hu et al., cancer cells exhibited a higher proportion of bound FAD as the concentration of pyruvate in the media was increased, which is consistent with our findings.^37^

The metabolic behavior of two-dimensional cell cultures can significantly differ from one of the cells studied *in vivo* experiments. As a result, there is an interest in the investigations of 3D models, such as organoids, and spheroids that are closer to the *in vivo* settings.^25^^,^^26^^,^^38^^,^^39^ The use of 3D structures for drug screening may have a significant value in personalized chemotherapy in oncology. Compared with the previous studies on 3D models, we use a single wavelength excitation at 750 nm to excite both NAD(P)H and flavins and detect the fluorescence signal from both of the coenzymes with a multi-channel spectral detector. The use of combined spectral and fluorescence lifetime data allows us to observe both spectroscopic and fluorescence lifetime heterogeneity in 3D models. The cells in the spheroid’s shell have a higher proliferative activity, and therefore, are more glycolytic compared with the cells in the inner layers. The more oxidative metabolism of the cells in the core was displayed as a longer NAD(P)H mean lifetime, lower free/bound NAD(P)H ratio, and higher amplitude (i.e., emission intensity) of flavins. Others confirmed that such changes are associated with the shifts of cellular metabolism to an oxidative profile.^40^

Although spectroscopic FLIM is a nice approach to studying metabolism in cells and 3D models, it has several limitations. First, the IRF width of the system is ∼220  ps FWHM or 95 ps RMS. The IRF is much broader than the electrical IRF of the SPC-150 TCSPC/FLIM modules (6.8 ps FWHM) and broader than the response of FLIM systems with GaAsP hybrid detectors (100 ps) or ultra-fast hybrid detectors (19 ps).^17^ The broad IRF of the multi-wavelength system is due to the transit-time spread in the multi-channel PMT and thus unavoidable with the detectors available. Second, the current approach does not differentiate flavins neither by type (FAD or FMNs) nor by the conformation (stacked and unstacked). Finally, we have to note that the models that we use in our analysis do not allow us to differentiate NADH and NADPH. These limitations need to be addressed in further studies.

## 5 Conclusion

To conclude, we reported the approach to probe the metabolic state of cells in culture and spheroids using excitation at a single wavelength of 750 nm and a fluorescence time-resolved spectral detection with the consequent global analysis of the data. Such an approach not only simplifies image acquisition protocol but also allows us to disentangle the impacts of free NAD(P)H, bound NAD(P)H, and flavin components, evaluate changes in their fluorescence parameters (emission spectra and fluorescence lifetime) upon treating cells with metabolic inhibitors, and sense metabolic heterogeneity within 3D tumor spheroids. Our method could be used for other studies, for example, for the separation of fluorescence from endogenous and exogenous fluorophores. This could also be a major advantage when this assay is finally transferred to clinical applications in the cancer field.

## Supplementary Material



## Data Availability

Data underlying the results presented in this paper are not publicly available at this time but may be obtained from the authors upon reasonable request.
