# Supplementary material for: Simultaneous assessment of NAD(P)H and flavins with multispectral fluorescence lifetime imaging microscopy at a single excitation wavelength of 750 nm
Source: J Biomed Opt. 2024 Sep 30;29(10):106501. doi: 10.1117/1.JBO.29.10.106501 (PMC11440180; doi:10.1117/1.JBO.29.10.106501)
Supplement: Supplementary file 1 [file JBO_029_106501_SD001.docx]

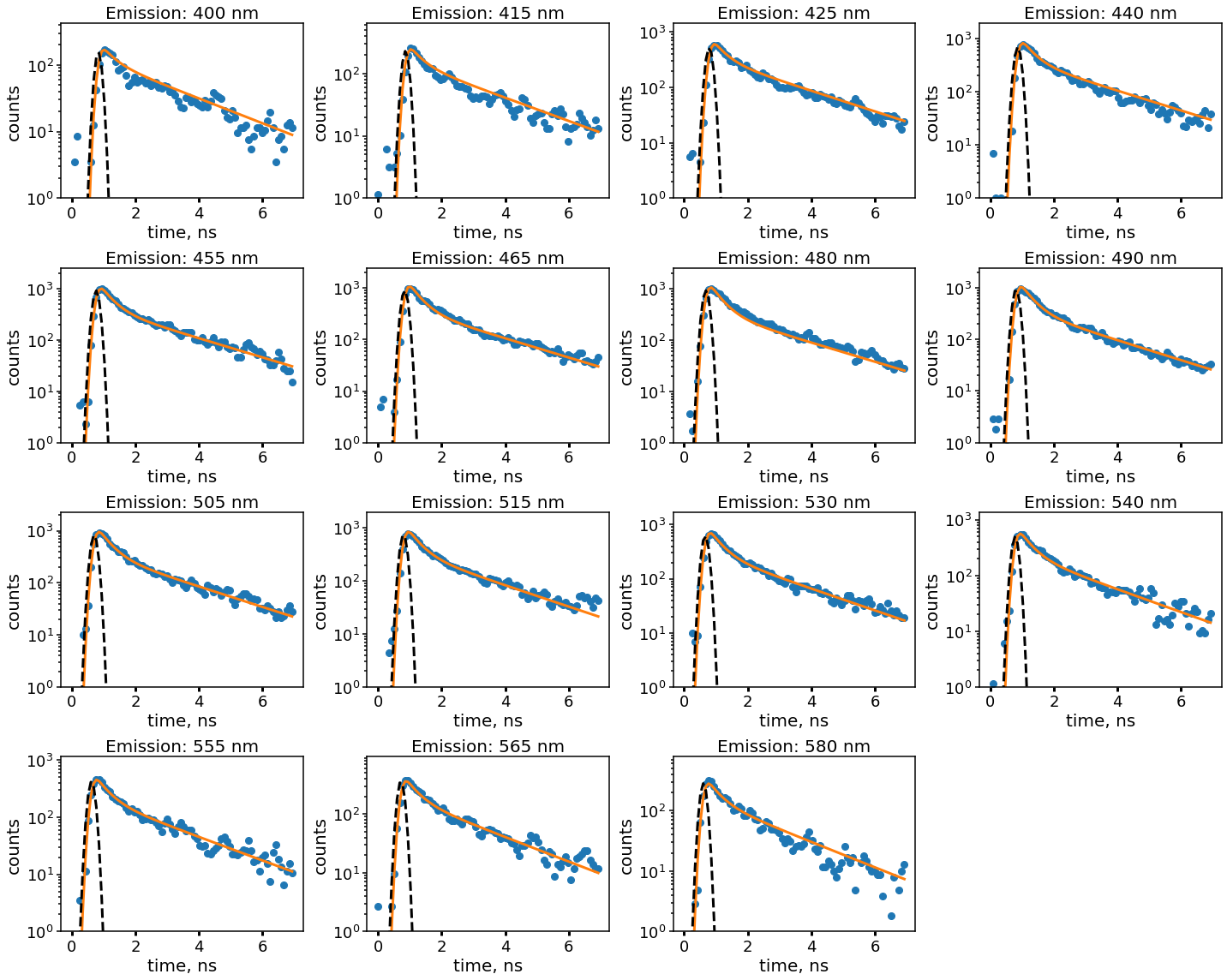


Fig. S1. Fluorescence decay curves and best fits for different emission wavelengths. The decay time of free NAD(P)H was fixed to 0.37 ns, while the lifetimes of bound NAD(P)H and flavins were fitted from the experimental data. Black dashed line corresponds to instrument response function.

Table S1. Fluorescence decay parameters obtained using the developed global approximation model for decay curves presented in Figure S1.

| $\tau_{free}^{NAD(P)H}$, ns | $a_{free}^{NAD(P)H}$, % | $\tau_{bound}^{NAD(P)H}$, ns | $a_{bound}^{NAD(P)H}$, % | $\tau_{flavins}$, ns | $a_{flavins}$, % |
| --- | --- | --- | --- | --- | --- |
| 0.37 (fixed) | 65.7 ± 0.7 | 2.29 ± 0.03 | 24.5 ± 0.6 | 1.81 ± 0.05 | 9.8 ± 1.1 |

Table S2. Amplitudes of the component 3( $a_{3}(\lambda))$, responsible for the fluorescence parameters of flavins at different emission wavelengths λ_em_ for exemplary fluorescence decay curves presented in Figure S1.

| λ_em_,nm | 400 - 490 | 490 | 505 | 515 | 530 | 540 | 555 | 565 | 580 |
| --- | --- | --- | --- | --- | --- | --- | --- | --- | --- |
| $a_{3}{(\lambda)}/{a_{flavins}}$ | 0.0 (fixed) | 0.53 | 0.76 | 1 | 0.88 | 0.84 | 0.79 | 0.77 | 0.63 |


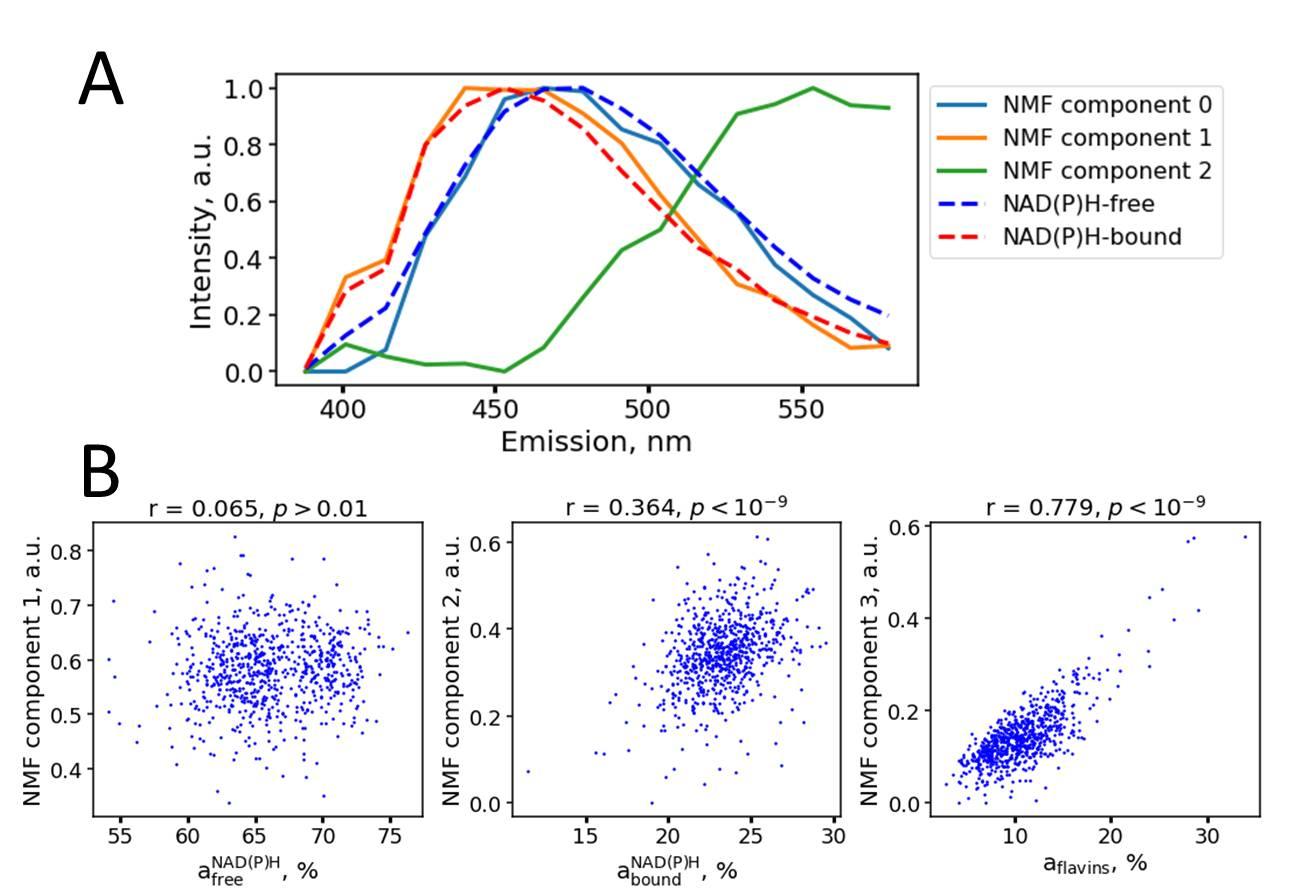


Fig. S2. Decomposition using non-negative matrix factorization (NMF) of: (A) Time-averaged fluorescence emission spectra; (B) Time-resolved fluorescence emission spectra.


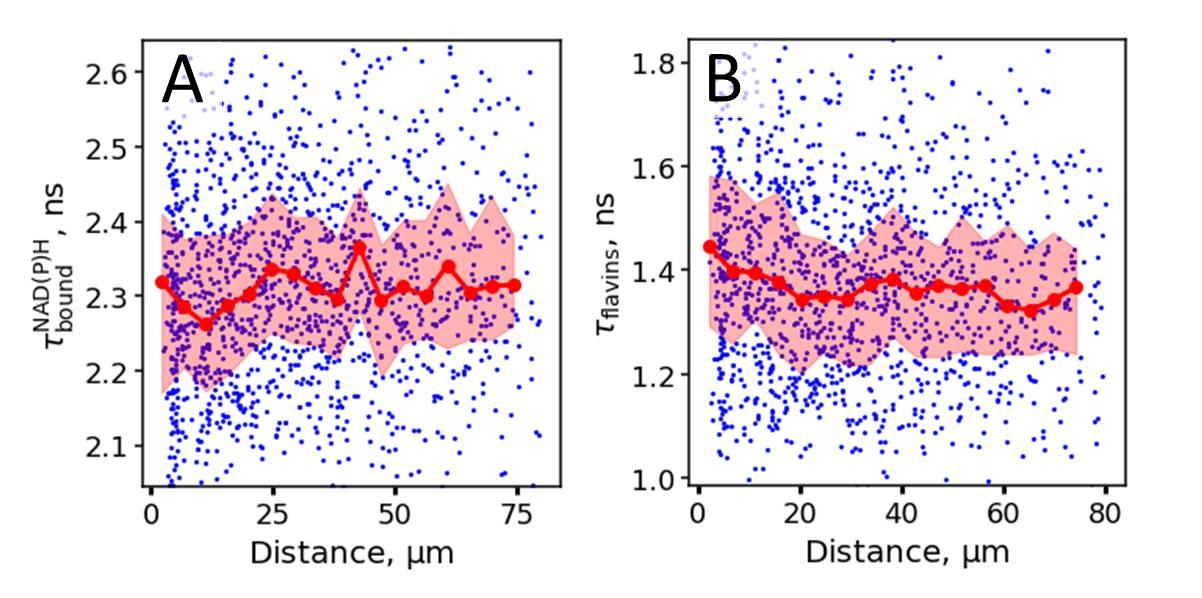


Fig. S3. The spatial distribution of the mean fluorescence lifetimes in the spheroids. Distance is measured from the surface of a spheroid. Blue dots are the fluorescence lifetime values for the specific singe cells. Red line displays the averaged value of the fluorescence lifetime and the red shadow area is the standard deviation from the average value. (A) For NAD(P)H; (B) for flavins.
